# Clinicopathological and Prognostic Significance of Long Non-coding RNA MIAT in Human Cancers: A Review and Meta-Analysis

**DOI:** 10.3389/fgene.2021.729768

**Published:** 2021-09-30

**Authors:** Yongfeng Wang, Liangyin Fu, Tingting Lu, Guangming Zhang, Jiawei Zhang, Yuanbin Zhao, Haojie Jin, Kehu Yang, Hui Cai

**Affiliations:** ^1^The First Clinical Medical College of Gansu University of Chinese Medicine (Gansu Provincial Hospital), Lanzhou, China; ^2^General Surgery Clinical Medical Center, Gansu Provincial Hospital, Lanzhou, China; ^3^Key Laboratory of Molecular Diagnostics and Precision Medicine for Surgical Oncology in Gansu Province, Gansu Provincial Hospital, Lanzhou, China; ^4^Evidence-Based Medicine Center, School of Basic Medical Sciences, Lanzhou University, Lanzhou, China; ^5^Institution of Clinical Research and Evidence Based Medicine, Gansu Provincial Hospital, Lanzhou, China; ^6^Second Clinical Medical College, Lanzhou University, Lanzhou, China; ^7^The First Clinical Medical College of Lanzhou University, Lanzhou, China; ^8^Key Laboratory of Evidence Based Medicine and Knowledge Translation of Gansu Province, Lanzhou, China

**Keywords:** lncRNA, MIAT, cancer, prognosis, meta-analysis

## Abstract

**Background:** Although the treatment of cancer has made evident progress, its morbidity and mortality are still high. A tumor marker is a critical indicator for early cancer diagnosis, and timely cancer detection can efficiently help improve the prognosis of patients. Therefore, it is necessary to identify novel markers associated with cancer. LncRNA myocardial infarction associated transcript (MIAT) is a newly identified tumor marker, and in this study, we aimed to explore the relationship between MIAT and clinicopathological features and patient prognosis.

**Methods:** We searched PubMed, Embase, Web of Science, and The Cochrane Library from inception to September 2020 to identify correlational studies. Then, we extracted valid data and used Stata software to make forest plots. We used the hazard ratio (HR) or odds ratio (OR) with 95% CI to evaluate the relationship between aberrant expression of MIAT and patients' prognosis and clinicopathological features.

**Results:** The study included 21 studies, containing 2,048 patients. Meta-analysis showed that overexpression of lncRNA MIAT was associated with poor overall survival (OS) (HR = 1.60, 95% CI, 1.31–1.96, *p* < 0.001). In addition, high expression of MIAT could forecast tumor size (OR = 2.26, 95% CI 1.34–3.81, *p* = 0.002), distant metastasis (OR = 2.54, 95% CI 1.84–3.50, *p* < 0.001), TNM stage (OR = 2.38, 95% CI 1.36–4.18, *p* = 0.002), lymph node metastasis (OR = 2.59, 95% CI 1.25–5.36, *p* = 0.011), and the degree of differentiation (OR = 2.65, 95% CI 1.54–4.58, *p* < 0.001). However, other clinicopathological features, including age (OR = 1.07, 95% CI 0.87–1.32, *p* = 0.516), gender (OR = 0.95, 95% CI 0.77–1.19, *p* = 0.668), and histology (OR = 0.72, 95% CI 0.48–1.10, *p* = 0.128) were not significantly different from high expression of MIAT.

**Conclusions:** Our study showed that overexpression of MIAT is related to poor overall survival and clinicopathological features. MIAT can be considered a novel tumor marker to help diagnose tumors earlier and improve patient prognosis.

## Introduction

With high incidence and mortality rates, cancer has been a threat to global human health (Wu et al., 2016). Since most cancer patients are diagnosed in an advanced stage, various human cancers have a low 5-year survival rate (Siegel et al., 2017). In a previous article, 18.1 million new cancer cases and 9.6 million cancer deaths were reported to occur in 2018 worldwide (Bray et al., 2018). Therefore, it is critical to identify novel tumor markers for diagnosis, prognosis, and tumor treatment.

Long non-coding RNA (lncRNA) are endogenous transcripts, longer than 200 nucleotides, which lack the specific open reading frame, resulting in the disability of encoding proteins (Xue et al., 2017). LncRNA can regulate diverse physiological and pathological processes via post-transcriptional, post-translational, and epigenetic mechanisms. Growing evidence suggests that lncRNAs often play critical biological functions in gene expression, transcription, cellular development, differentiation, proliferation, and cell fate (Binabaj et al., 2019; Ahn and Kim, 2020). Moreover, studies have shown that lncRNAs with high specificity and accuracy can become biomarkers in cancers (Qian et al., 2020). Therefore, lncRNA can be viewed as potential tumorigenic and antitumorigenic RNA (Huarte and Rinn, 2010). Due to their specific expression and functional diversity in various cancer types, lncRNA has promising cancer diagnosis applications, prognosis, and therapeutic effects.

Myocardial infarction associated transcript (MIAT, also commonly known as GOMAFU, LINC00066, or RNCR2) is a lncRNA that was first identified as a locus associated with myocardial infarction susceptibility (Ishii et al., 2006). In a previous study, it was reported that upregulation of MIAT could result in microvascular dysfunction by enhancing endothelial cell proliferation and migration (Yan et al., 2015). MIAT knockdown decreased cell viability, migration, invasion, and tumor cell cycle arrest in the G1 phase (Zhang et al., 2020a). Abnormal expression of MIAT has been observed in multiple malignancies, and it appears related to the pathogenesis of several cancers including breast cancer (BC) (Li et al., 2018a), cervical cancer (CC) (Zhang et al., 2020b), cholangiocarcinoma (CCA) (Chang et al., 2020), renal cell carcinoma (RCC) (Qu et al., 2018), colorectal cancer (CRC) (Liu et al., 2018), esophageal cancer (EC) (Zhang et al., 2020a), gastric cancer (GC) (Sha et al., 2018), hepatocellular carcinoma (HCC) (Huang et al., 2018), lung cancer (LC) (Lin et al., 2020), and pancreatic cancer (PC) (Li et al., 2018b).

There are no integrated data to clarify the relationship between lncRNA MIAT and cancer prognosis. Furthermore, there has been controversy on the predictive value of lncRNA MIAT expression in different types of cancer (Liu et al., 2020; Zhang et al., 2020b). Moreover, many studies that published on MIAT have acquired unique results attributing to limitations, such as a small sample size. Therefore, we collected all eligible studies concerning MIAT and tested the association between MIAT expression and overall survival (OS) and clinicopathological significance in different types of cancer through comprehensive meta-analysis.

## Materials and Methods

### Registration

The study was registered on PROSPERO (the registration number is: CRD42021228343).

### Search Strategy

We performed a comprehensive search of PubMed, the Cochrane Library, EMBASE, and Web of Science restricted to English-language articles up to September 2020. The search terms were used as follows: (“MIAT” OR “GOMAFU” OR “RNCR2” OR “Myocardial infarction associated transcript” OR “long non-coding RNA MIAT” OR “long non-coding RNA MIAT” OR “lncRNA MIAT”) AND (“cancer” or “carcinoma” or “sarcoma” or “melanoma” or “tumor” or “neoplasm” or “adenoma”).

### Inclusion and Exclusion Criteria

The inclusion criteria were as follows: (1) lncRNA MIAT expression in cancer tissues; (2) patients were divided into two groups based on lncRNA MIAT expression levels; (3) the study provided at least one of the following clinical outcomes: OS, poor histological (differentiation) grade, earlier distant metastasis, high tumor stage and lymph node metastasis, and the number of patients with larger tumor size; (4) sufficient data were extracted to compute the hazard ratio (HR) and 95% CI of survival or the odds ratio (OR) and 95% CI of clinicopathological parameters. The exclusion criteria were as follows: (1) publication letters, case reports, reviews, conference abstracts, etc.; (2) studies without clinical features; (3) studies unrelated to lncRNA MIAT.

### Data Extraction

Two authors independently evaluated and extracted relevant information (Li et al., 2016; Pan et al., 2018). A consensus was reached by the third author in a situation of a disagreement (Yao et al., 2016; Peinemann et al., 2019). According to the inclusion and exclusion criteria, the following information was collected: (1) the surname of the first author, year of publication, and country of origin; (2) tumor type, specimen, and sample size; (3) lncRNA MIAT assessment technique; (4) cut-off value and follow-up time; (5) OR of clinical parameters, including age, gender, tumor size, distant metastasis, lymph node metastasis, TNM stage, and histological (differentiation) grade; (6) survival outcome (HR with 95% CI).

### Quality Assessment

The quality of the literature was evaluated according to the vital checklist recommended by the Newcastle–Ottawa Scale (NOS) Dutch Cochrane Center. This scale uses nine entries to assess the study, and 1 score was earned for satisfying an item. The total score was between 0 and 9. NOS scores of ≥7 represented high-quality study results.

### Data Synthesis and Statistical Analysis

Some of the included studies had accurate survival data, and these dates could directly be used. When the article only provided Kaplan–Meier (KM) curves without accurate survival data, Engauge Digitizer V.4.1 software was used to extract the data from the survival curves and the HR and 95% CI were calculated (Parmar et al., 1998). We collected the pooled HRs and their 95% CIs from the included studies. Moreover, we used both the log HR and the SE to aggregate the survival outcome while applying the OR and corresponding 95% CI to summarize clinicopathological parameters. Furthermore, inter-study heterogeneity was assessed by the χ^2^ test together with the *I*^2^ statistic. The *p*-value of Q test (*PQ*) <0.05 and *I*^2^ > 50% indicated statistical heterogeneity among studies, and the random-effects model was adopted when analyzing the results. In other cases, the fixed-effect model was employed. We used forest plots to display the meta-analysis results and assessed any prospective bias in the publication using Egger's test. Analyses were conducted with Stata 12.0 (Stata, College Station, TX, USA) for Windows, and *p* < 0.05 was considered statistically significant.

### Target Gene Prediction and Signal Pathway Network Construction

We downloaded lncRNA MIAT associated genes from the MEM-Multi Experiment Matrix database. Subsequently, we performed Gene Ontology (GO) and the Kyoto Encyclopedia of Genes and Genomes (KEGG) pathway enrichment analysis. And then, we used Cytoscape to construct and visualize a signaling pathway network.

## Results

### Characteristics of Studies

**Figure 1:**
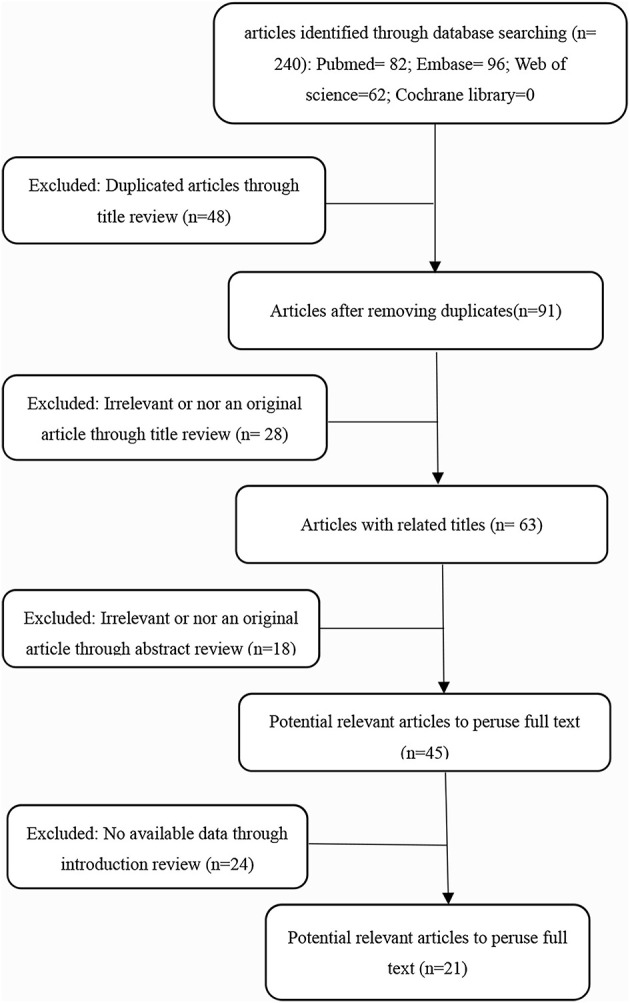
Flow diagram of this meta-analysis.

In this meta-analysis, 21 studies (Lai et al., 2017; Fu et al., 2018, 2019; Li et al., 2018b, 2020a,b; Qu et al., 2018; Sha et al., 2018; Shao et al., 2018; Lin et al., 2019; Wang et al., 2019a,b; Zhang et al., 2019, 2020b; Zhao et al., 2019; Zhong et al., 2019; Liu et al., 2020; Wu et al., 2020; Xu et al., 2020; Zhou et al., 2020; Zhu et al., 2020) were selected from 240 articles initially searched. Figure 1 shows our screening process and results, according to the PRISMA guidelines. This study involved 2,047 patients, all of whom were from China. The minimum sample size of each study was 24, and the maximum was 448. The publication year was from 2017 to 2020, and all articles were published in the English language.

The enrolled studies were composed of 14 types of cancers, including breast cancer (*n* = 1) (Li et al., 2020a), cervical cancer (*n* = 2) (Liu et al., 2020; Zhang et al., 2020b), renal cell carcinoma (*n* = 1) (Qu et al., 2018), gastric cancer (*n* = 2) (Sha et al., 2018; Xu et al., 2020), leukemia (*n* = 1) (Wang et al., 2019a), lung cancer (*n* = 6) (Lai et al., 2017; Fu et al., 2018; Lin et al., 2019; Li et al., 2020b; Wu et al., 2020; Zhou et al., 2020), melanoma (*n* = 1) (Zhu et al., 2020), multiple myeloma (*n* = 1) (Fu et al., 2019), osteosarcoma (*n* = 1) (Zhang et al., 2019), ovarian cancer (*n* = 1) (Shao et al., 2018), pancreatic cancer (*n* = 1) (Li et al., 2018b), papillary thyroid cancer (*n* = 1) (Wang et al., 2019b), tongue squamous cell carcinoma (*n* = 1) (Zhong et al., 2019), and Wilms' tumor (*n* = 1) (Zhao et al., 2019). OS was reckoned as a survival outcome, referring to 67% (14/21) among these studies. The main essential characteristics of these studies are shown in Table 1.

**Table 1:** Characteristics of the included studies.

**Cancer**	**First author**	**Year**	**Country**	**Sample type**	**Sample size (n)**	**Detection method**	**Cut-off value**	**Survival analysis**	**Hazard ratios**	**NOS score**
Breast cancer (Li et al., 2020a)	Dezhi Li	2020	China	Tissue	84	qRT-PCR	NR	OS	K-M curve	6
Cervical cancer (Zhang et al., 2020b)	Lei Zhang	2020	China	Tissue	64	qRT-PCR	NR	NR	NR	6
Cervical cancer (Liu et al., 2020)	Yanbin Liu	2020	China	Tissue	24	qRT-PCR	Median value	OS	K-M curve	8
RCC (Qu et al., 2018)	Yan Qu	2018	China	Tissue	448	qRT-PCR	Median value	OS	K-M curve	8
GC (Sha et al., 2018)	Min Sha	2018	China	Tissue	120	qRT-PCR	Average value	NR	NR	8
GC (Xu et al., 2020)	Hao Xu	2020	China	Tissue	109	qRT-PCR	Median value	OS	K-M curve	7
Leukemia (Wang et al., 2019a)	Gaoyan Wang	2019	China	Tissue	121	qRT-PCR	Median value	OS	K-M curve	8
LC (Fu et al., 2018)	Yunfeng Fu	2018	China	Tissue	212	qRT-PCR	Median value	OS	K-M curve	8
LC (Lin et al., 2019)	D. Lin	2019	China	Tissue	60	qRT-PCR	Median value	OS	K-M curve	8
LC (Lai et al., 2017)	I-Lu Lai	2017	China	Tissue	60	qRT-PCR	Average value	NR	NR	8
LC (Wu et al., 2020)	Longqiu Wu	2020	China	Tissue	60	qRT-PCR	Median value	OS	K-M curve	8
LC (Zhou et al., 2020)	Zhi Zhou	2020	China	Tissue	80	qRT-PCR	NR	OS	K-M curve	7
LC (Li et al., 2020b)	Fannian Li	2020	China	Tissue	58	qRT-PCR	NR	NR	NR	7
Melanoma (Zhu et al., 2020)	Lifei Zhu	2020	China	Tissue	90	qRT-PCR	NR	NR	NR	7
MM (Fu et al., 2019)	Yunfeng Fu	2019	China	Tissue	123	qRT-PCR	Median value	OS	K-M curve	8
Osteosarcoma (Zhang et al., 2019)	Chunyan Zhang	2019	China	Tissue	27	qRT-PCR	Average value	NR	NR	7
OC (Shao et al., 2018)	Shiqing Shao	2018	China	Tissue	53	qRT-PCR	Median value	OS	K-M curve	8
PC (Li et al., 2018b)	Ting-Fu Li	2018	China	Tissue	38	qRT-PCR	Median value	OS	K-M curve	8
PTC (Wang et al., 2019b)	Renjie Wang	2019	China	Tissue	50	qRT-PCR	Median value	NR	NR	7
TSCC (Zhong et al., 2019)	Waisheng Zhong	2019	China	Tissue	116	qRT-PCR	Median value	OS	K-M curve	8
Wilms' tumor (Zhao et al., 2019)	X.-S. Zhao	2019	China	Tissue	50	qRT-PCR	Median value	OS	K-M curve	8

*RCC, renal cell carcinoma; GC, gastric cancer; LC, lung cancer; MM, multiple myeloma; OC, ovarian cancer; PC, pancreatic cancer; PTC, papillary thyroid cancer; TSCC, tongue squamous cell carcinoma; qRT-PCR, quantitative real time polymerase chain reaction; NR, not reported*.

### Association Between the MIAT Expression Level and OS

**Figure 2:**
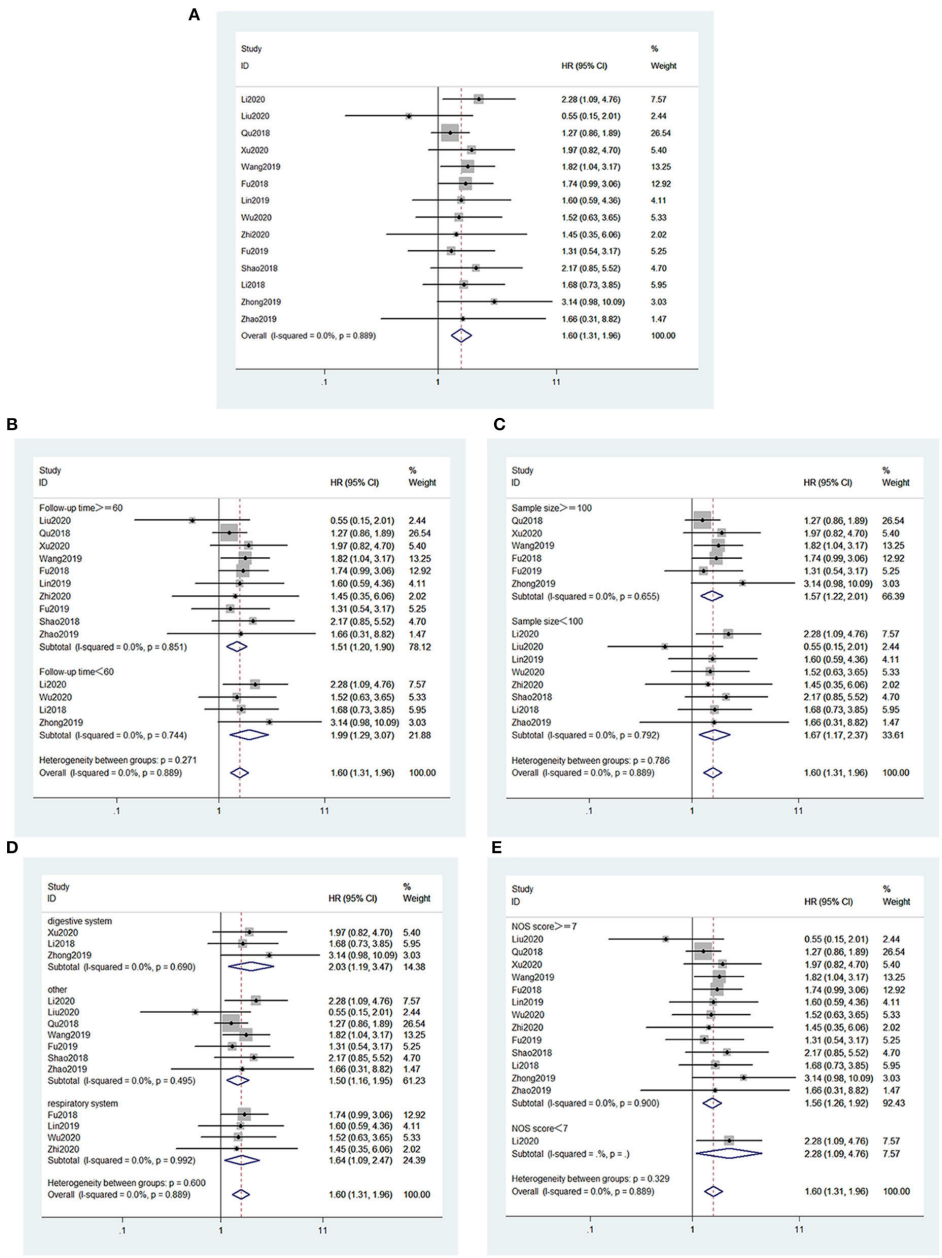
Forest plots for the association of MIAT expression with overall survival and subgroup analysis of MIAT expression with overall survival. **(A)** Forest plots for the association of MIAT expression with overall survival. **(B)** Subgroup analysis stratified by follow-up time. **(C)** Subgroup analysis stratified by sample size. **(D)** Subgroup analysis stratified by type of cancer. **(E)** Subgroup analysis stratified by NOS score.

Figure 2 presents the association between OS and MIAT. There were 14 studies (Fu et al., 2018, 2019; Li et al., 2018b, 2020a; Qu et al., 2018; Shao et al., 2018; Lin et al., 2019; Wang et al., 2019a; Zhao et al., 2019; Zhong et al., 2019; Liu et al., 2020; Wu et al., 2020; Xu et al., 2020; Zhou et al., 2020), consisting of 1,578 patients who claimed a connection between the OS of cancer patients and MIAT expression levels. The fixed effect model was applied (*I*^2^ = 0.0%, *PQ* = 0.889). A pooled HR = 1.60 was found in the analysis (95% CI, 1.31–1.96, *p* < 0.001; Figure 2A). Patients with lower survival rates increased dramatically among the high MIAT expression group. Thus, these findings demonstrated that MIAT served as an independent element for the survival rate in patients with malignancies. In addition, we performed subgroup analysis to investigate the association between MIAT expression levels and the OS according to the following factors: follow-up time (≥60 or <60 months) (Figure 2B), sample size (≥100 or <100 tissues) (Figure 2C), type of cancer (digestive system, respiratory system or other) (Figure 2D), and article quality (NOS scores ≥7 or <7) (Figure 2E). Results of the subgroup analysis did not alter the predictive value of MIAT for OS in these cancers.

### Association Between MIAT and Clinicopathological Features

**Figure 3:**
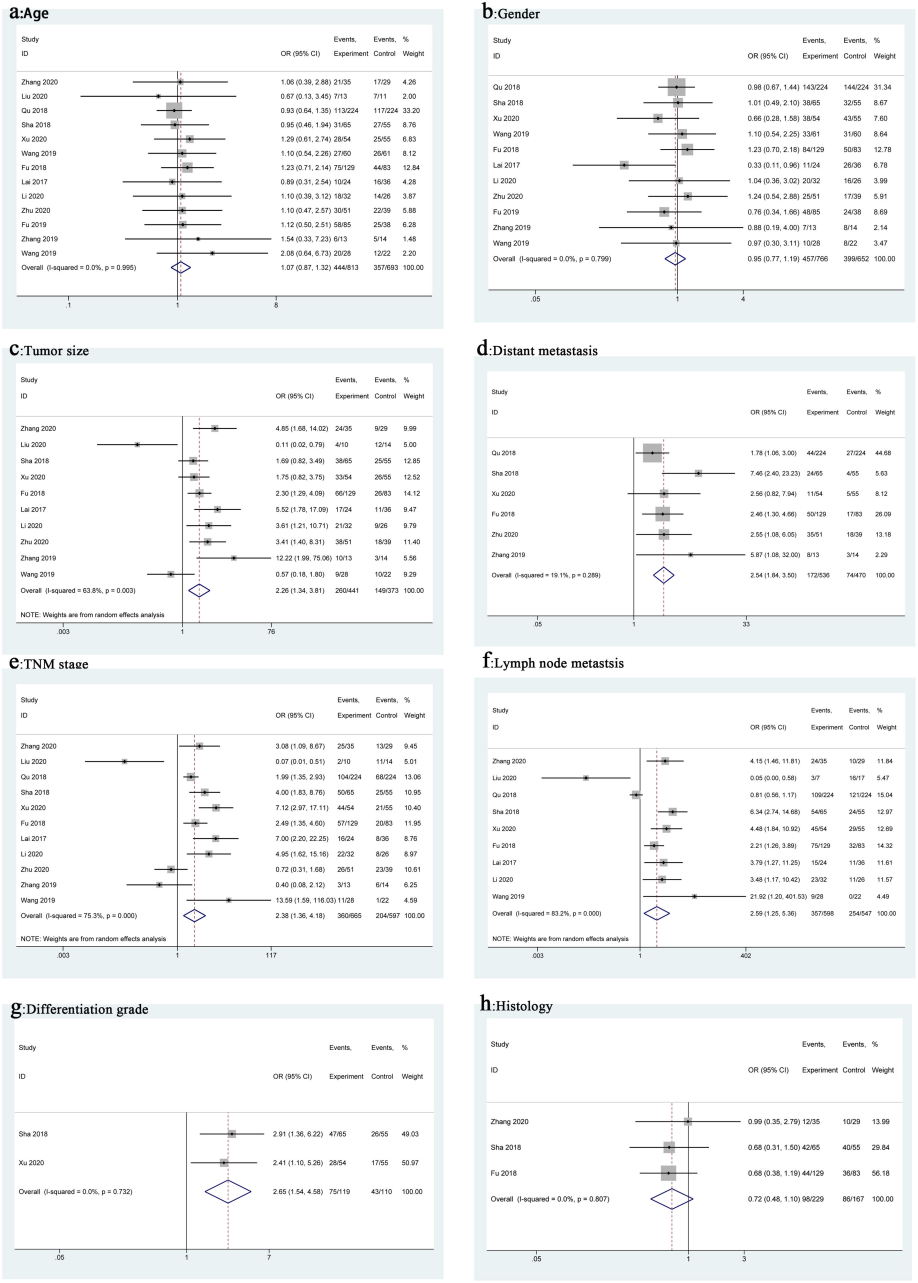
Forest plots for association of MIAT expression with clinicopathological features: Age **(a)**, Gender **(b)**, Tumor size **(c)**, Distant metastasis **(d)**, TNM stage **(e)**, Lymph node metastasis **(f)**, Differentiation grade **(g)**, Histology **(h)**.

We used ORs and the 95% CIs to study the relationship between the MIAT expression level and clinicopathological features. These analysis results are shown in Figure 3 and Table 2. From the pooled ORs, high MIAT expression significantly correlated with tumor size (OR = 2.26, 95% CI 1.34–3.81, *p* = 0.002, Figure 3C), distant metastasis (OR = 2.54, 95% CI 1.84–3.50, *p* < 0.001, Figure 3D), TNM stage (OR = 2.38, 95% CI 1.36–4.18, *p* = 0.002, Figure 3E), lymph node metastasis (OR = 2.59, 95% CI 1.25–5.36, *p* = 0.011, Figure 3F), and differentiation grade (OR = 2.65, 95% CI 1.54–4.58, *p* < 0.001, Figure 3G). However, no significant correlation was observed between MIAT expression and age (OR = 1.07, 95% CI 0.87–1.32, *p* = 0.516, Figure 3A), gender (OR = 0.95, 95% CI 0.77–1.19, *p* = 0.668, Figure 3B), and histology (OR = 0.72, 95% CI 0.48–1.10, *p* = 0.128, Figure 3H).

**Table 2:** Association of MIAT expression with clinicopathological features.

**Clinicopathological parameters studies (n)**	**Patients (n)**	**OR (95% CI)**	***P*-value**	**Heterogeneity (*I*^**2**^, *P*)**	**Model**
Age (elderly vs. non-elderly)	1,506	1.07 (0.87, 1.32)	0.516	0.0%, 0.995	Fixed
Gender (male vs. female)	1,418	0.95 (0.77, 1.19)	0.668	0.0%, 0.799	Fixed
Tumor size (large size vs. small size)	814	2.26 (1.34, 3.81)	0.002	63.8%, 0.003	Random
Distant metastasis (presence vs. absence)	1,006	2.54 (1.84, 3.50)	<0.001	19.1%, 0.289	Fixed
TNM stage (III + IV vs. I + II)	1,262	2.38 (1.36, 4.18)	0.002	75.3%, <0.001	Random
Lymph node metastasis (positive vs. negative)	1,145	2.59 (1.25, 5.36)	0.011	83.2%, 0.000	Random
Differentiation grade (poorly and moderately vs. well)	229	2.65 (1.54, 4.58)	<0.001	0.0%, 0.732	Fixed
Histology (adenocarcinoma vs. other types)	396	0.72 (0.48, 1.10)	0.128	0.0%, 0.807	Fixed

### Publication Bias and Sensitivity Analysis

**Figure 4:**
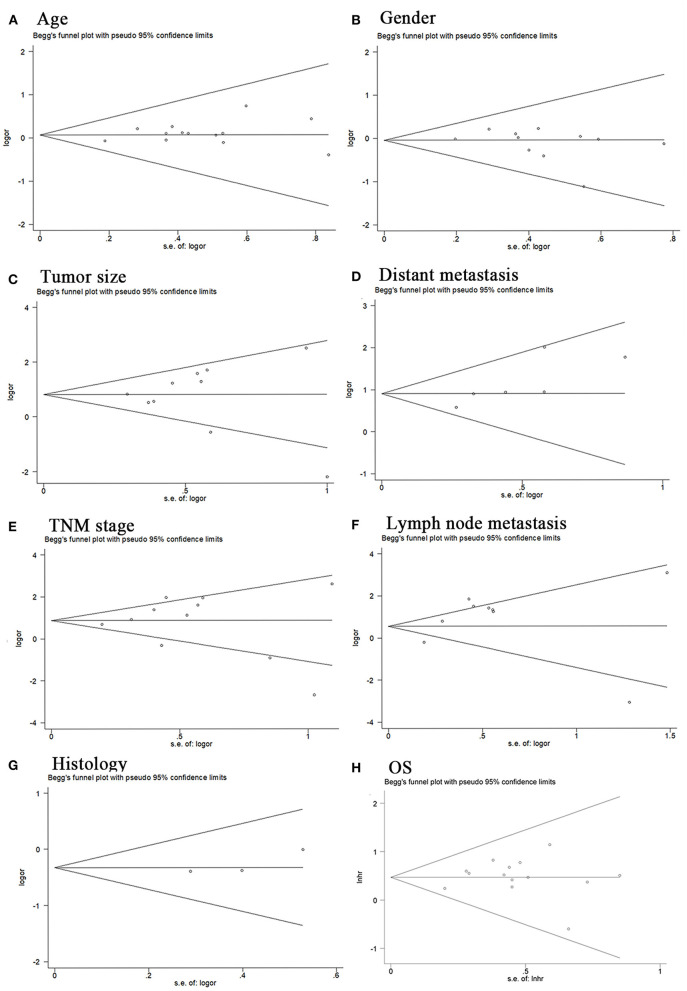
Begg's publication bias plots. Age **(A)**, Gender **(B)**, Tumor size **(C)**, Distant metastasis **(D)**, TNM stage **(E)**, Lymph node metastasis **(F)**, Histology **(G)**, OS **(H)**.

**Figure 5:**
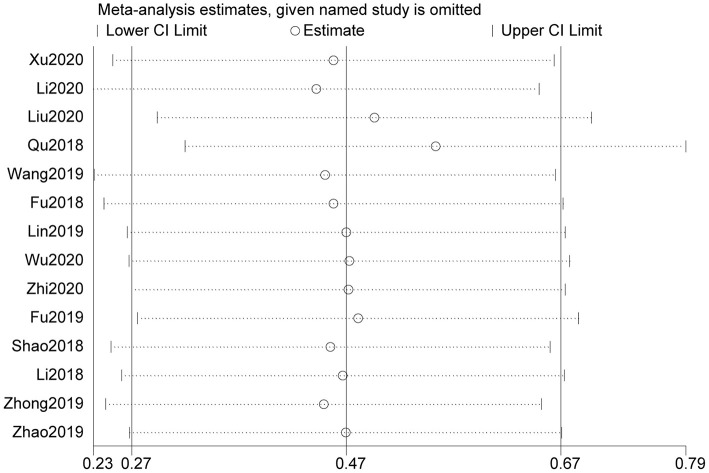
Sensitivity analysis for studies about OS by omitting each study sequentially.

The Begg test was performed to explore possible publication bias. Our data showed that the Begg plots did not show significant publication bias for age (*P*>|t| =0.246; Figure 4A), gender (*P*>|t| = 0.27; Figure 4B), tumor size (*P*>|t| = 0.883; Figure 4C), distant metastasis (*P*>|t| = 0.05; Figure 4D), TNM stage (*P*>|t| = 0.961; Figure 4E), lymph node metastasis (*P*>|t| = 0.118; Figure 4F), histology (*P*>|t| = 0.335; Figure 4G), and OS (*P*>|t| = 0.60; Figure 4H). Furthermore, we conducted sensitivity analysis to estimate the impact of independent studies on the overall results of OS. After excluding each eligible study, the results did not change significantly, thereby confirming the robustness of the conclusions of the meta-analysis (Figure 5). Therefore, our pooled results of MIAT expression on prediction of OS were reliable.

### Analysis of MIAT-Related Genes

**Figure 6:**
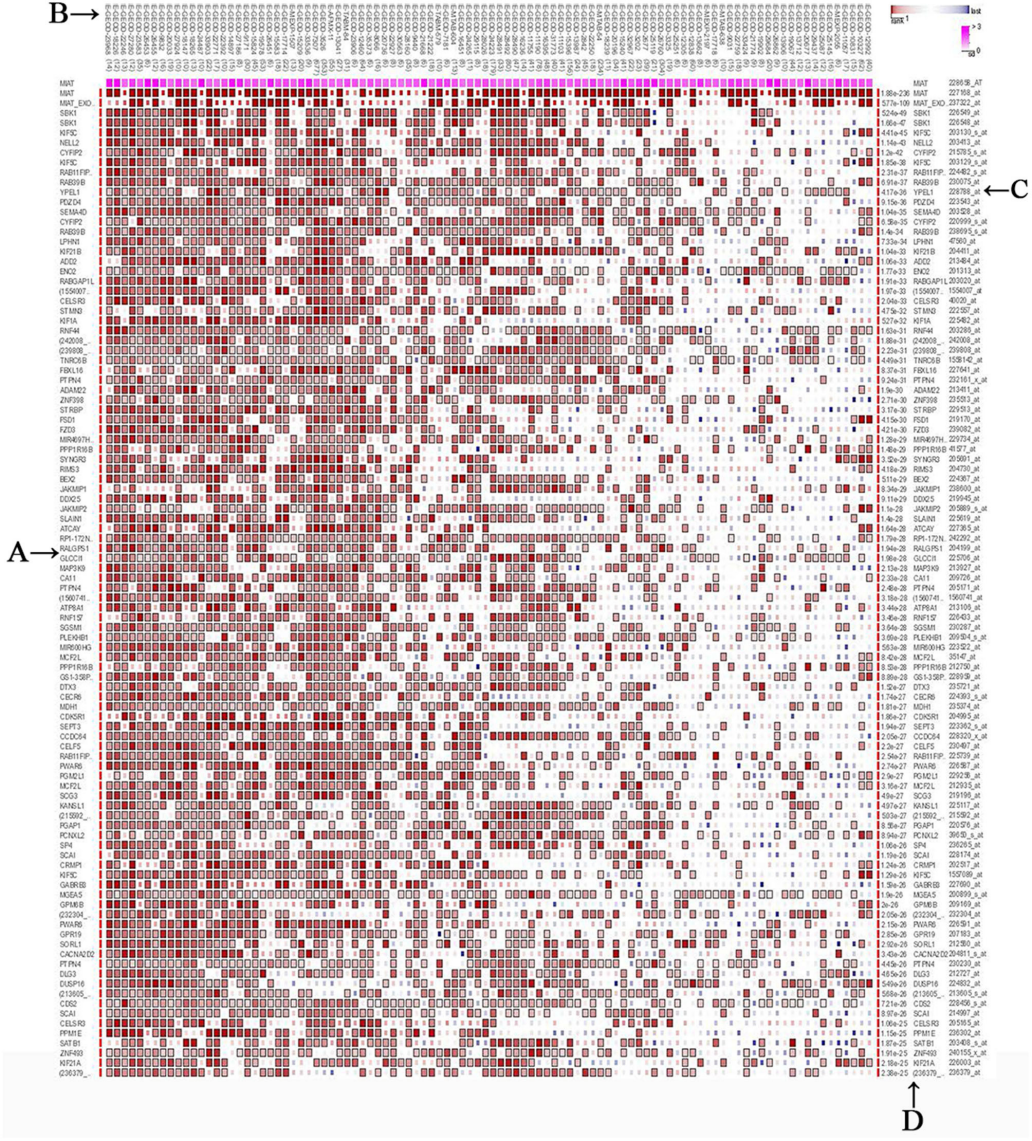
The top 200 predicted target genes of lincRNA MIAT by using Multi Experiment Matrix (MEM, http://biit.cs.ut.ee/mem/) website. **(A)** Predicted target genes; **(B)** single experimental data set; **(C)** gene probes; **(D)** *P* values.

**Figure 7:**
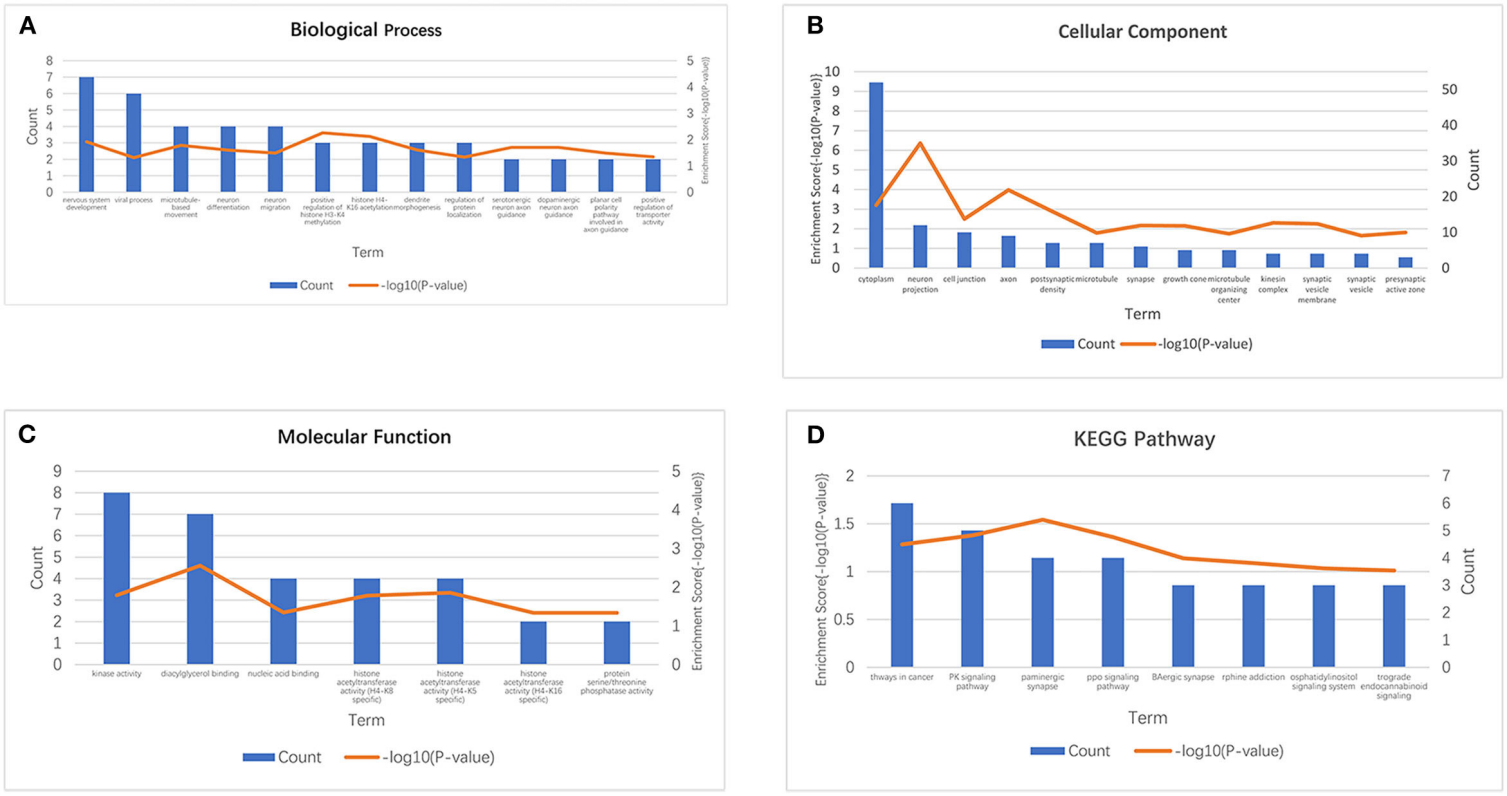
GO terms and the KEGG pathway. **(A)** GO enrichment of target genes in biological process ontology (*P* < 0.05). **(B)** GO enrichment of target genes in cellular component ontology (*P* < 0.05). **(C)** GO enrichment of target genes in molecular function ontology (*P* < 0.05). **(D)** The top 8 pathways related to the differentially expressed genes by the KEGG database analysis. BP, biological process; CC, cellular component; GO, gene ontology analysis; KEGG, Kyoto Encyclopedia of Genes and Genomes; MF, molecular function.

**Figure 8:**
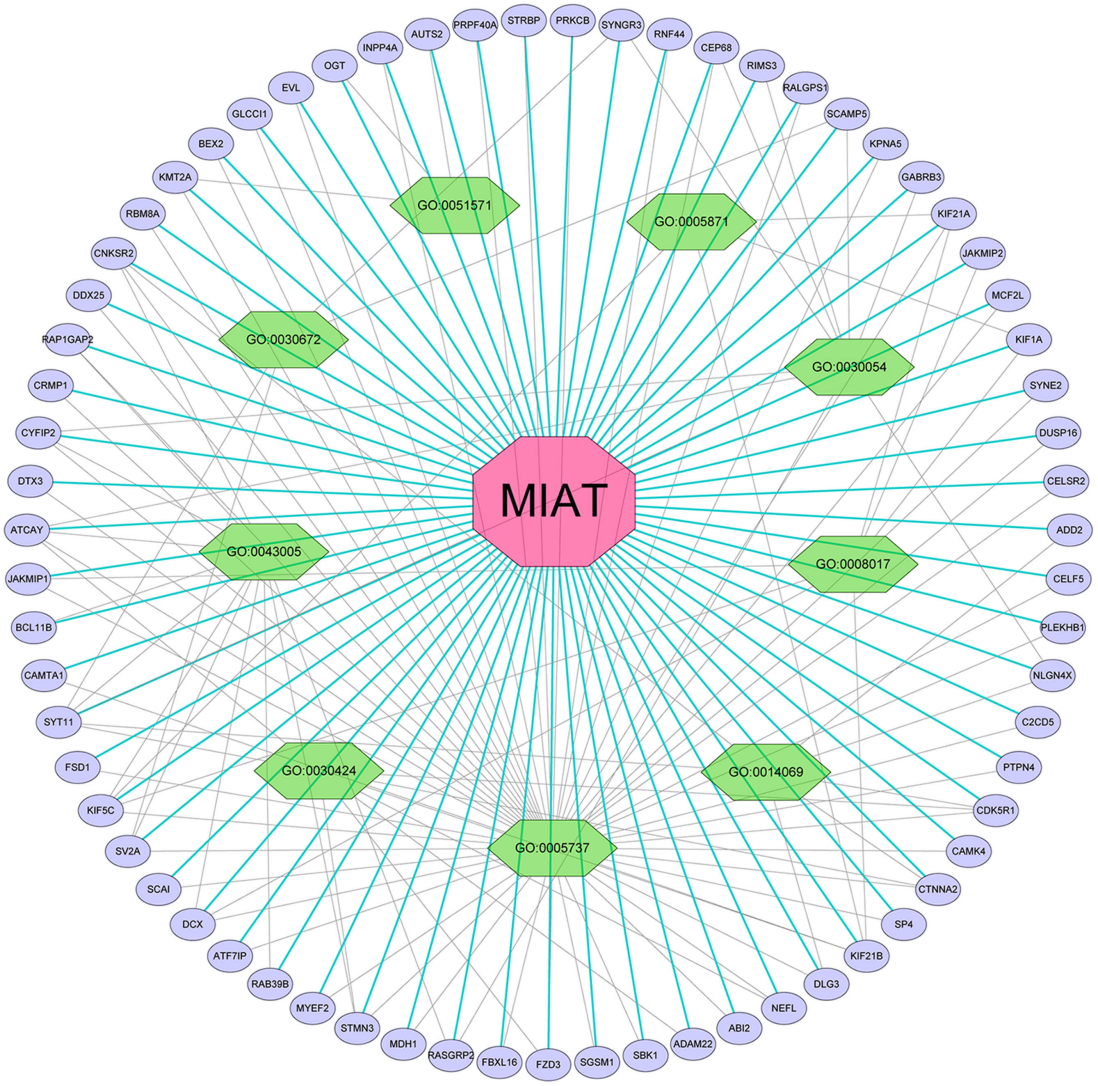
Differentially expressed gene interaction network analysis. Purple nodes represent target genes and green nodes represent the related pathway. As indicated in red, MIAT localized at the center of the network.

We screened the top 200 co-expressed genes of MIAT from MEM-Multi Experiment Matrix database and analyzed their correlations (Figure 6). Based on the *p*-value, we discovered that MIAT, MIAT_EXON5_1, and SBK1 ranked as the top three different predicted target genes, which showed that they were related to lncRNA MIAT gene expression. To further understand the underlying molecular mechanisms involved, we next analyzed the GO and KEGG pathways (Figure 7; Table 3). Furthermore, we constructed a signaling pathway network by using Cytoscape (Figure 8).

**Table 3:** Gene ontology analysis of lncRNA MIAT-related genes.

**GO number**	**Description**	**Genes**	***P*-value**
GO:0043005	Neuron projection	CNKSR2, RAP1GAP2, CYFIP2, ATCAY, BCL11B, SYT11, KIF5C, SV2A, DCX, RAB39B, STMN3, RASGRP2	4.38E−07
GO:0030424	Axon	FZD3, ATCAY, SYT11, ADAM22, NEFL, STMN3, KIF21B, CTNNA2, CDK5R1	1.05E−04
GO:0005737	Cytoplasm	CYFIP2, C2CD5, PLEKHB1, CELF5, STMN3, CELSR2, DUSP16, SYNE2, ATCAY, KIF5C, MCF2L, NEFL, KIF21A, KPNA5, KIF21B, RALGPS1, CEP68, RNF44, PRKCB, STRBP, PRPF40A, INPP4A, CNKSR2, DCX, EVL, GLCCI1, BEX2, KMT2A, RBM8A, DDX25, CRMP1, DTX3, JAKMIP1, SV2A, CAMTA1, FSD1, CTNNA2, SCAI, ATF7IP, RAP1GAP2, FZD3, MYEF2, MDH1, FBXL16, SGSM1, SBK1, ABI2, DLG3, SP4, CAMK4, PTPN4, CDK5R1	6.29E−04
GO:0014069	Postsynaptic density	CNKSR2, NLGN4X, DLG3, SYT11, CTNNA2, CDK5R1, ADD2	0.0013
GO:0008017	Microtubule binding	KIF5C, DCX, KIF21A, KIF1A, KIF21B, JAKMIP1, JAKMIP2	0.0028
GO:0030054	Cell junction	GABRB3, CYFIP2, CEP68, SCAMP5, ATCAY, RIMS3, NLGN4X, SYT11, SYNGR3, RASGRP2	0.0032
GO:0005871	Kinesin complex	KIF5C, KIF21A, KIF1A, KIF21B	0.0050
GO:0051571	Positive regulation of histone H3-K4 methylation	AUTS2, KMT2A, OGT	0.0055
GO:0030672	Synaptic vesicle membrane	SCAMP5, SYT11, SV2A, SYNGR3	0.0056

## Discussion

Human health is seriously threatened by cancer. Although the detection and treatment of cancers have significantly progressed, a study showed a gradual increase in the incidence of cancer in recent years (Sung et al., 2021). It is essential to detect and treat earlier to improve the prognosis of cancer patients. The discovery of lncRNA has led to a new understanding of cancer. Accumulating studies have shown that lncRNA is aberrantly expressed in a broad spectrum of cancers and can impact tumor occurrence and development (Bhan et al., 2017; Lin and Yang, 2018). For example, in a study by Wu et al. (2021), it was found that lncRNA SNHG1 is overexpressed in ovarian cancer and affects ovarian cancer proliferation and metastasis. They demonstrated that SNHG1 plays a vital role in tumor progression and may be a valuable marker for ovarian cancer prognosis. Therefore, identifying novel tumor markers related to the prognosis of malignant tumors is critical (Mattiuzzi and Lippi, 2019). LncRNA can be used as a molecular marker of cancer and has the potential to monitor and diagnose tumors, which is of great significance for the prognosis and treatment of cancer.

In recent years, accumulating studies have demonstrated aberrant expression of lncRNA MIAT in many cancers, including breast cancer (Li et al., 2020a), cervical cancer (Liu et al., 2020; Zhang et al., 2020b), renal cell carcinoma (Qu et al., 2018), gastric cancer (Sha et al., 2018; Xu et al., 2020), leukemia (Wang et al., 2019a), lung cancer (Lai et al., 2017; Fu et al., 2018; Lin et al., 2019; Li et al., 2020b; Wu et al., 2020; Zhou et al., 2020), melanoma (Zhu et al., 2020), myeloma (Fu et al., 2019), osteosarcoma (Zhang et al., 2019), ovarian cancer (Shao et al., 2018), pancreatic carcinoma (Li et al., 2018b), papillary thyroid cancer (Wang et al., 2019b), tongue squamous cell carcinoma (Zhong et al., 2019), and Wilms' tumor (Zhao et al., 2019). In this meta-analysis, lncRNA MIAT was upregulated in cancer tissue, except for the study by Liu et al. (2020), which showed a downregulation. Because the function of MIAT in different cancers is still controversial and remains clarified, we conducted this meta-analysis to evaluate the clinicopathological significance and prognostic value of aberrant expression of MIAT in cancer patients.

Our meta-analysis showed that high expression of lncRNA MIAT was associated with poor OS. In addition, we analyzed the relationship between high expression of MIAT and clinicopathological features. We found that high expression of MIAT was associated with tumor size, distant metastasis, TNM stage, lymph node metastasis, and degree of differentiation. However, high expression of MIAT was not significantly correlated with age, gender, and OR histology. We conclude that high expression of MIAT is correlated with poor prognosis and the clinicopathological features of cancer patients, and that MIAT is a potential predictor of poor prognosis of cancer.

Although many studies showed that MIAT serves as an important prognostic factor for patients with various tumors, the underlying systems of how MIAT impacts cancer are still unknown. Thus, further mechanistic studies showed that overexpression of MIAT could significantly promote cancer growth and metastasis. Knockdown of MIAT could significantly inhibit cell proliferation, invasion, apoptosis, and the progress of the cancerization process. In breast cancer, MIAT inhibition upregulated DLG3 and activated the Hippo signaling pathway to suppress proliferation and promote apoptosis of breast cancer cells (Li et al., 2020a). It has been demonstrated that MIAT is a novel potential therapeutic target for breast cancer. In addition, MIAT also is highly expressed in gastric cancer. Knockdown of MIAT can inhibit the growth and metastasis of gastric cancer. MIAT promotes the proliferation, migration, and invasion of cancer cells by regulating DDX5 (Sha et al., 2018). MIAT was upregulated in renal cell carcinoma tissues and promoted cancer proliferation and metastasis by competitively binding miR-29c. Moreover, to further explore the relation between MIAT and more cancers, we summarized MIAT and its functional roles and related genes in Table 4.

**Table 4:** Summary of lncRNA MIAT functional roles and related genes.

**Cancer**	**Expression**	**Functional role**	**Related genes**	**References**
Breast cancer	Upregulate	Cell proliferation and apoptosis	DLG3	Li et al., 2020a
Cervical cancer	Upregulate/ downregulate	Cell proliferation and migration	PI3K/Akt/mTOR miR-150-5p, CDKN1B	Liu et al., 2020; Zhang et al., 2020b
Renal cell carcinoma	Upregulate	Cell proliferation and migration	miR-29c, Loxl2	Qu et al., 2018
Gastric cancer	Upregulate	Cell proliferation and migration	miR-141/DDX5	Sha et al., 2018; Xu et al., 2020
Leukemia	Upregulate	Cell proliferation and apoptosis	miR-495	Wang et al., 2019a
Lung cancer	Upregulate	Cell migration, invasion, proliferation, metastasis	miR-34a, miR-1246, MMP9, miR-184, miR-149-5p, FOXM1, miR-128-3p/PELI3	Lai et al., 2017; Fu et al., 2018; Lin et al., 2019; Li et al., 2020b; Wu et al., 2020; Zhou et al., 2020
Melanoma	Upregulate	Cell proliferation, invasion, and EMT	miR-150	Zhu et al., 2020
Myeloma	Upregulate	Cell proliferation and apoptosis	miR-29b	Fu et al., 2019
Osteosarcoma	Upregulate	Cell proliferation, migration, invasion, and apoptosis	miR-128-3p/VEGFC	Zhang et al., 2019
Ovarian cancer	Upregulate	Cell proliferation and apoptosis	miR-330-5p	Shao et al., 2018
Pancreatic carcinoma	Upregulate	Cell proliferation and metastasis	miR-133	Li et al., 2018b
Papillary thyroid cancer	Upregulate	Cell proliferation, migration, and invasion	miR-212	Wang et al., 2019b
Tongue squamous cell carcinoma	Upregulate	EMT	Wnt/β-catenin	Zhong et al., 2019
Wilms' tumor	Upregulate	Cell migration and invasion	DGCR8	Zhao et al., 2019

To further determine the role of lncRNA MIAT, we carried out target gene prediction and signaling pathway analysis on the lncRNA MIAT by using the MEM-Multi Experiment Matrix database. Moreover, GO and KEGG analyses were performed. The results of our investigation showed that MIAT, MIAT_EXON5_1, and SBK1, which play essential roles in multiple tumors, were significantly related to lncRNA MIAT gene expression. The outcomes of GO and KEGG pathway analysis demonstrated that lncRNA MIAT was significantly related to the cytoplasm, cell junction, and chromatin binding activity. For the KEGG pathway, lncRNA MIAT was significantly related to cancer-related pathways.

However, there are several limitations to the current analysis. First, some HRs were calculated according to the KM curve, and calculating HRs and corresponding 95% CIs from the survival curve may not be accurate enough. Second, the eligible studies were all performed in China, so it is unclear whether our results are suitable for other countries. Third, different studies have inconsistent definitions of cut-off values for the expression of MIAT; no subgroup analysis was conducted to test whether cut-off values were factors that affect the analysis. In addition, our study does not address the ability of MIAT to predict risk as an independent risk factor. Finally, some data comes from online databases. To better determine the clinical application value of MIAT, these findings need to be further confirmed in an enormous scope of the sample.

In summary, this meta-analysis demonstrated that high expression of lncRNA MIAT was closely related to the poor OS of cancer patients. In addition, differentially expressed MIAT could act as oncogenes or tumor suppressors to improve cancer diagnosis, discover potential treatment targets, and improve prognosis. Furthermore, more high-quality studies with a large sample size are required to further certify the prognostic value of lncRNA MIAT in cancers.

## Data Availability Statement

The original contributions presented in the study are included in the article/supplementary material, further inquiries can be directed to the corresponding author/s.

## Author Contributions

YW conceived the study. JZ and YZ performed the literature search. LF and GZ extracted the required data. TL performed the statistical analyses. YW and LF wrote a draft. KY and HC reviewed the paper. All authors contributed to the article and approved the submitted version.

## Funding

This study was supported by the Natural Science Foundation of Gansu Province, China (No. 18JR3RA052); National Scientific Research Project Cultivation Plan of Gansu Provincial People's Hospital (No. 2019-206); Lanzhou Talent Innovation and Entrepreneurship Project Task Contract (No. 2016-RC-56); National Key Research and Development Program (No. 2018YFC1311500); Fundamental Research Funds for the Central Universities (No. 2020jbkyzx001; lzujbky-2020-kb20); and 2021 Graduate Innovation Fund Project of Gansu University of Traditional Chinese Medicine, No. 2021CX54.

## Conflict of Interest

The authors declare that the research was conducted in the absence of any commercial or financial relationships that could be construed as a potential conflict of interest.

## Publisher's Note

All claims expressed in this article are solely those of the authors and do not necessarily represent those of their affiliated organizations, or those of the publisher, the editors and the reviewers. Any product that may be evaluated in this article, or claim that may be made by its manufacturer, is not guaranteed or endorsed by the publisher.
